# Harmonizing and Contrasting Psychotherapy Approaches: Case Presentation

**DOI:** 10.1192/j.eurpsy.2024.108

**Published:** 2024-08-27

**Authors:** S. Tanyeri Kayahan

**Affiliations:** Psychiatry, Turkish Republic Ministry of Health Yalvaç State Hospital, Isparta, Türkiye

## Abstract

**Abstract:**

Various psychotherapeutic approaches could be used for the assessment, formulation, and treatment of the same case. It is of great importance to include cultural evaluation and empathetic reformulation in psychotherapy applications. This case presentation illustrates a female patient in her thirties who was consulted at the psychiatric outpatient clinic with depressive symptomatology, which presented after a psychosocially stressful life event. Following a detailed clinical evaluation, psychotherapeutic treatment was planned with the diagnosis of major depressive disorder. In this case presentation and subsequent discussion, the case-specific advantages, along with standard and differing aspects of different psychotherapeutic methods and treatments, will be evaluated.

**Disclosure of Interest:**

None Declared

